# From NTM (*Nontuberculous mycobacterium*) to *Gordonia bronchialis*—A Diagnostic Challenge in the COPD Patient

**DOI:** 10.3390/diagnostics12020307

**Published:** 2022-01-25

**Authors:** Monika Franczuk, Magdalena Klatt, Dorota Filipczak, Anna Zabost, Paweł Parniewski, Robert Kuthan, Lilia Jakubowska, Ewa Augustynowicz-Kopeć

**Affiliations:** 1 Respiratory Physiopathology Department, National Tuberculosis and Lung Diseases Research Institute, 01-138 Warsaw, Poland; 2 Microbiology Department, National Tuberculosis and Lung Diseases Research Institute, 01-138 Warsaw, Poland; m.klatt@igichp.edu.pl (M.K.); d.filipczak@igichp.edu.pl (D.F.); a.zabost@igichp.edu.pl (A.Z.); e.kopec@igichp.edu.pl (E.A.-K.); 3 Institute of Medical Biology, Polish Academy of Sciences, 90-001 Lodz, Poland; pparniewski@cbm.pan.pl; 4 Chair and Department of Medical Microbiology, Medical University of Warsaw, 02-091 Warsaw, Poland; rkuthan@wum.edu.pl; 5 Radiology Department, National Tuberculosis and Lung Diseases Research Institute, 01-138 Warsaw, Poland; lilia.jakubowska@icloud.com

**Keywords:** *Gordonia bronchialis*, microbiological diagnostics, respiratory infection

## Abstract

In patients with chronic obstructive pulmonary disease, respiratory infections are of various aetiology, predominantly viral and bacterial. However, due to structural and immunological changes within the respiratory system, such patients are also prone to mycobacterial and other relatively rare infections. We present the 70-year old male patient with chronic obstructive pulmonary disease (COPD) and coexisting bronchial asthma, diagnosed due to cough with purulent sputum expectoration lasting over three months. The first microbiological investigation of the sputum sample revealed the growth of mycobacteria. The identification test based on protein MPT64 production indicated an organism belonging to NTM *(nontuberculous mycobacterium*). However, further species identification by genetic testing verified the obtained culture as not belonging to the Mycobacterium genus. Based on observed morphology, the new characterisation identified an aerobic actinomycete, possibly a *Nocardia* spp. The isolated strain was recultured on standard microbiological media. The growth of colonies was observed on Columbia blood agar plates and solid Löewenstein-Jensen medium. The Gram and Zhiel-Nielsen stains revealed the presence of Gram-positive acid-fast bacilli. The extraction protocol and identification were performed in two repetitions; the result was *G. bronchialis*, with a confidence value of 99% and 95%, respectively. The gene sequencing method was applied to confirm the species affiliation of this isolate. The resulting sequence was checked against the 16S ribosomal RNA sequences database (Bacteria and Archaea). The ten best results indicated the genus Gordonia (99.04–100%) and 100% similarity of the 16S sequenced region was demonstrated for *Gordonia bronchialis*. The case described indicates that the correct interpretation of microbiological test results requires the use of advanced microbiology diagnosis techniques, including molecular identification of gene sequences. From a clinical point of view, *Gordonia bronchialis* infection or colonization may present a mild course, with no febrile episodes and no significant patient status deterioration and thus, it may remain undiagnosed more often than expected.

## 1. Introduction

In patients with chronic obstructive pulmonary disease, respiratory infections are of various aetiology, predominantly viral and bacterial. However, due to structural and immunological changes within the respiratory system, such patients are also prone to nontuberculous mycobacterial pulmonary disease and other rare infections. The relatively rare species *G. bronchialis* has been recognised as an etiological factor for respiratory infection, although it was identified for the first time in sputum cultures obtained from patients with bronchiectasis and cavitary tuberculosis [1]. Despite many years since the first case report, the available data on the *G. bronchialis* infection or colonization, the prevalence and diagnostical procedures remain very scarce. Thus, the diagnosis and effective treatment of patients with such infection remain a challenge.

## 2. Case Presentation

We present a 70-year old male patient, with a diagnosis of chronic obstructive pulmonary disease (COPD) and coexisting bronchial asthma, a former smoker for 15 years, with previous exposure of about 43 pack-years. The patient had been taking prescribed appropriate inhaled treatment: salmeterol + fluticasone 500 mcg 2 × 1 puff (in DPI, dry powder inhaler), tiotropium 18 mcg once a day (Respimat, soft mist liquid inhaler), and salbutamol (pMDI, pressurized metered-dose inhaler) per need as a relief medication.

He was referred to a specialist due to a cough with purulent sputum expectoration lasting for over three months. Five months before the consultation, the patient had experienced a respiratory infection, with a fever reaching 40 °C, cough, purulent expectoration, and dyspnea. Due to suspicion of pneumonia, the general practitioner decided to apply an oral trimethoprim-sulfamethoxazole therapy. Unfortunately, the medication caused severe dyspnea and rash and was withdrawn immediately. No further alternative antibiotic treatment was implemented.

At the presentation, the patient was afebrile, with no chills, night sweats, and weight loss within the last three months. However, discrete dry rales and rattling sounds on auscultation were present on physical examination.

In spirometry, he presented moderately severe airway obstruction and no significant improvement after short-acting β2-agonist bronchodilation agent was found: FEV1%FVC 51%, FEV1 1.76 L 50%; post bronchodilation FEV1 1.87 L 53%. Vital capacity (VC) remained within the normal limits: VC 4.0 L 90% of predicted.

The laboratory tests revealed slightly elevated neutrophils and monocytes count: neutrophils 5.27 × 10^9^ (70.0%, N 34.0–67.9%), monocytes 0.99 × 10^9^ (13.2%, N 5.3–12.2%), and decreased count of lymphocytes 1.02 × 10^9^ (13.6%, N 21.8–53.1%). The C-reactive protein remained within normal limits (0.4 < 5 mg/L). The biochemistry analysis showed slightly increased alanine transaminase (57 U/L; N < 44), and elevated lactate dehydrogenase LDH (527 U/L; N < 480). The immunological profile was also assessed: IgG 1230 IU/mL (700–1600), IgM 35 IU/mL (40–230), IgA 297 IU/mL (70–400), total IgE 333 IU/mL (<100).

**Figure 1:**
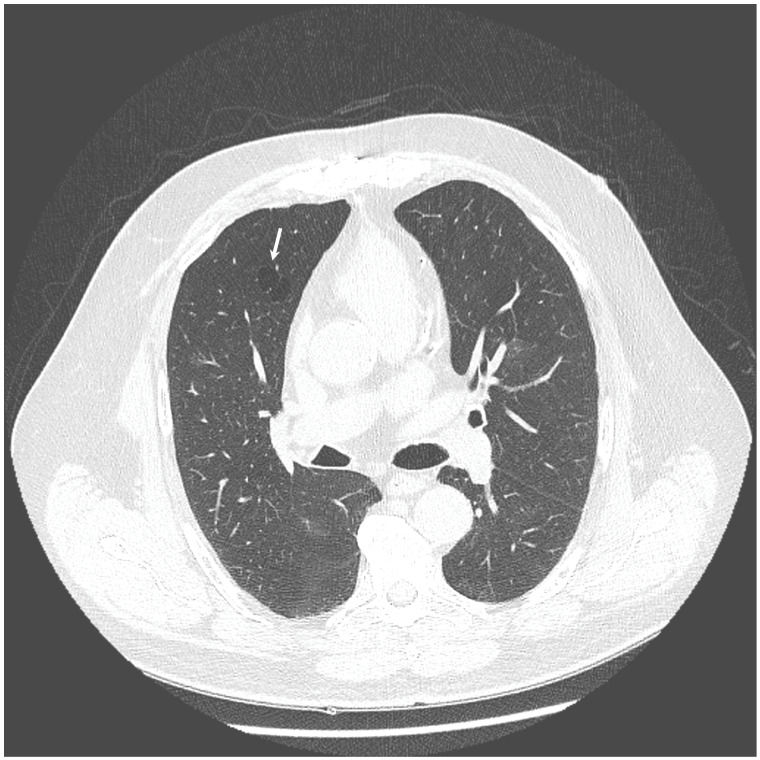
HRCT scan—Emphysema—the white arrow indicates the emphysematous changes in the lung.

**Figure 2:**
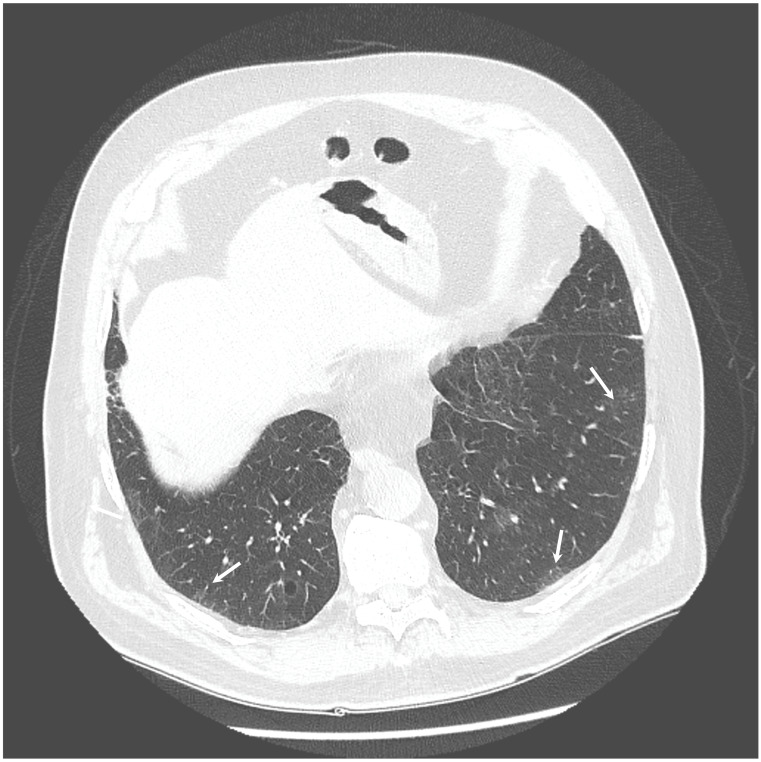
HRCT scan—Secretions accumulation and slight post-inflammatory changes (indicated by the white arrows).

The chest X-ray showed linear opacities in the lower right lobe due to atelectasis, comparable to the previous examination results. The high-resolution computed tomography confirmed emphysema features and revealed thickening of bronchial walls with secretions accumulation and slight post-inflammatory changes (Figure 1 and Figure 2). No features of bronchiectasis nor enlarged lymph nodes in mediastinum and hilar space were present, and no fluid signs in the pleura.

**Figure 3:**
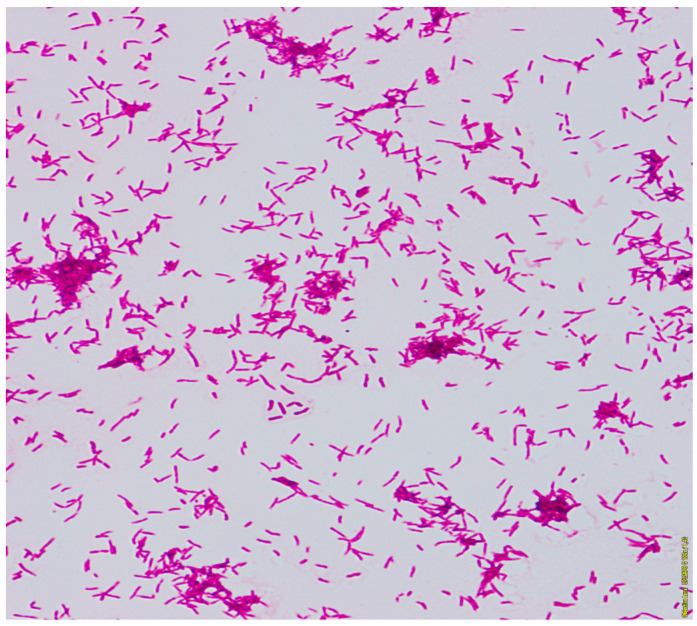
The acid-fast rods of mycobacterium. Smear made from a colony, Ziehl-Neelsen stain.

The general microbiological investigation did not reveal any aerobic bacteria growth. Three samples of the sputum were examined for tuberculosis and mycobacteria. From one of them, the growth on Middlebrook liquid medium in the Bactec MGIT system (Becton Dickinson BD, Sparks, MD, USA) after 12 days was obtained. The Zhiel-Neelsen staining of smear from pure culture revealed acid-fast mycobacteria (Figure 3).

**Figure 4:**
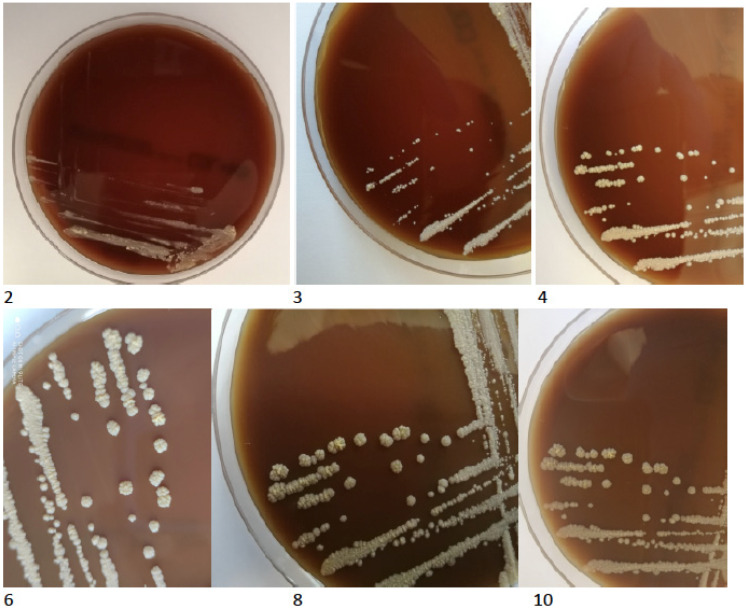
The growth of tiny creamy-yellowish colonies observed on Columbia blood agar plates (2, 3, 4, 6, 8, 10 days of incubation).

**Figure 5:**
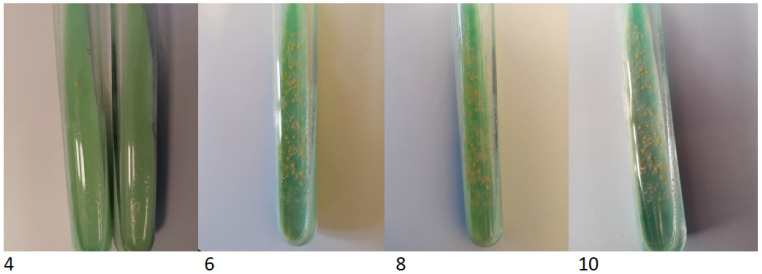
The colony growth on Löewenstein-Jensen solid medium on the 4th, 6th, 8th and 10th day of incubation.

The TBC ID MGIT (BD) identification test based on protein MPT64 production was performed, and the organism was preliminarily identified as NTM. However, further species identification by genetic test (GenoType Mycobacterium CM VER 2.0; Hain Lifescience, Nehren, Germany) verified the obtained culture as not belonging to the Mycobacterium genus. Based on observed morphology, the new characterisation identified an aerobic actinomycete, possibly a *Nocardia* spp. The isolated strain was re-cultured on standard microbiological media (Oxoid, Hampshire, UK), including solid Löewenstein-Jensen, Columbia agar with 5% sheep erythrocytes, McConkey agar, esculin medium, Sabouraud agar, chocolate agar and liquid Schaedler medium. All the cultures for bacteria were incubated at 37 °C for five days and 30 °C for seven days—medium for fungi. After 48 h of incubation, the growth of tiny creamy-yellowish colonies was observed on Columbia blood agar plates (Figure 4). After 4 days, the growth of yellow-orange colonies was noted on Löewenstein-Jensen solid medium (Figure 5).

No growth of microorganisms was found in the remaining cultures during incubation. The Gram and Zhiel-Nielsen stains revealed, respectively, the presence of Gram-positive rods and acid-fast bacilli. Unfortunately, the isolated microorganism was not identified to the genus level nor by biochemical method, nor by Matrix-Assisted Laser Desorption Ionization Time-of-Flight Mass Spectrometry (MALTI-TOF MS) with the standard protocol or with the use of a formic acid extraction protocol. Finally, the protocol for Mycobacterium and Nocardia identification was applied with the following modifications: initial extraction step, as described by the manufacturer, bacterial mass suspended in 70% ethanol, vortexed in the presence of glass bead, and extracted with formic acid has been changed—10 μL inoculation loop of bacteria colony was transferred into a 1.5 mL Eppendorf tube with 100 μL trifluoroacetic acid (TFA, Sigma, Saint Louis, MO, USA) and incubated at 37 °C for 30 min. In the next step, acetonitrile (Sigma) 1:1 (*v*/*v*) was added. The sample was centrifuged for 2 min at 9000 rpm. From the obtained supernatant, 1.5 μL was used for analysis on a Vitek MS system (Biomerieux, Durham, NC, USA). The extraction protocol and identification were performed in two repetitions; the result was *G. bronchialis*, with a confidence value of 99% and 95%, respectively.

## 3. Gene Sequencing

To confirm the species affiliation of this isolate, the V3-V4 region of the 16S rRNA gene was amplified with the use of primer “forward” 5′-ACTCCTACGGGAGGCAGCAG-3′ and primer “reverse” 5′-TACCAGGGTATCTAATCC-3′. The PCR was optimized and performed in a total volume of 25 μL consisting of 200 ng of DNA, 1× DreamTaq polymerase reaction buffer (includes 20 mM MgCl_2_) (Life Technologies, Carlsbad, CA, USA), 1 U DreamTaq polymerase (Life Technologies), 1 mM of each deoxynucleotide, 6% dimethyl sulfoxide (DMSO) and 0.4 μM of each primer. Reactions were performed using a Veriti™ 96-Well Thermal Cycler (Thermo Fisher Scientific, Waltham, MA, USA) under the following conditions: an initial denaturation step at 98 °C for 3 min, followed by 40 cycles of denaturation (98 °C for 10 s), annealing (51 °C for 20 s), extension (72 °C for 30 s) and final extension step (72 °C, 5 min). The 445 bp PCR amplicons were analyzed using horizontal 2% agarose gel electrophoresis at 70 V (2.4 V/cm) in a 1 × TAE buffer until the dye (bromophenol blue) reached 6 cm from the wells. The 100 bp Plus DNA size marker (Thermo Fisher Scientific) was used to normalize the size of each PCR product. The gel was stained in an ethidium bromide (EtBr) solution (0.5 μg/mL) for 10 min and destained in water for another 10 min. The gels were visualized under UV light using a FluorChem 8800 system with Alpha EaseFC v. 3.1.2 software (AlphaInnotech, San Leandro, CA, USA). The PCR products were purified using the EPPiC Fast mixture (A&A Biotechnology, Gdańsk, Poland), according to the manufacturer’s protocol. For sequencing of PCR products, the BrilliantDye™ Terminator v1.1 Cycle Sequencing Kit (NimaGen, Nijmegen, The Netherlands) was used, according to the manufacturer’s protocol. PCR products were purified with the use of BigDye XTerminator™ Purification Kit (Thermo Fisher Scientific), according to the manufacturer’s protocol and then sequenced using the 3500xl Genetic Analyzer (Applied Biosystems, San Diego, CA, USA). Sequencing results were analyzed using Chromas software version 2.4.1 (Technelysium, Brisbane, Australia). The resulting sequence was checked against the 16S ribosomal RNA sequences database (Bacteria and Archaea). The ten best results indicated the genus Gordonia (99.04–100%) and 100% similarity of the 16S sequenced region was demonstrated for *Gordonia bronchialis* DSM 43247 (accession NR_074529.1 and NR_027594.1) (Table 1).

While the microbiological investigation was being performed, the patient received intensive physiotherapy and bronchial drainage. As a result, the amount of sputum decreased significantly, and the patient improved remarkably. Unfortunately, as a result no material for further microbiological investigation was available. Finally, no other antimicrobial treatment was applied. Hence, he stayed under careful monitoring of a respiratory physician, and one year of follow-up revealed neither exacerbation nor respiratory infection.

## 4. Discussion

The first documented differentiation is known to have been published by Tsukamura in 1971 [1]. The author described a species isolated from the soil and patients with chronic respiratory diseases—bronchiectasis and cavitary tuberculosis.

*Gordonia* spp. are Gram-positive, nocardioform aerobic bacteria belonging to *Actinomycetales*, previously classified as *Rhodococcus* (*Nocardia*). They are ubiquitous in the soil and water environment and infrequently cause infections in humans. The main species of *Gordonia* are *G. terrae, G. bronchialis, G. sputi, G. polyisoprenivorans, G. otitis, G. arai* and *G. rubripertincta*. Thus, they are rare but most likely can cause human infections, both in immunocompromised and immunocompetent individuals.

Despite fifty years since the first case report, the available data on the *G. bronchialis* infection or colonization, the prevalence and diagnostical procedures are very scarce. Moreover, rarely have case reports been published in the literature concerning the issue of disorder variety. *Gordonia bronchialis* may cause various diseases, including sternal wound infections, respiratory and pleural infections [2], arthritis [3], cutaneous abscess related to needle injection or acupuncture [4,5], breast abscesses [6,7,8] and endophthalmitis [9]. The reported local infections were predominantly observed in immunocompetent individuals. Systemic infections were diagnosed mainly in patients with an underlying malignant disease, other immunocompromised conditions and chronic diseases like diabetes mellitus, cardiovascular diseases, autoimmune diseases, and sequestrated lung diagnosis [10,11,12]. Bacteremia, the most severe and emerging involvement, was contributed by a central venous catheter and other indwelling medical devices like peritoneal dialysis catheter or heart pacemaker [13,14,15,16,17]. The possible mechanism of such severe complication is the bacteria’s ability to form the biofilm by producing gordonan, an acidic polysaccharide inducing the cell aggregation, and adhesive properties to the hydrophobic surfaces, like catheters or other medical indwelling devices [18].

The sternal wound infection after open-heart surgery, mentioned above, was one of the most commonly reported local manifestations of the infection caused by *Gordonia bronchialis* [19,20,21,22]. The authors described hospital outbreaks and case series, following open-heart surgery, as a result of intraoperative transmission from a health care worker. Most of the presented patients had just local symptoms—erythema, swelling, pain and slight purulent exudation from the surgical wound. Moreover, the disease developed years after the coronary artery bypass surgery had been performed. Therefore, the diagnosis and detection of the causative factor required intensive and meticulous epidemiological and microbiological investigation in all reported cases. The clinical course of the infection in all patients showed nonneutropenic fever. In every case, a combination of two antibiotics on long-term therapy lasting over four weeks was applied with a favourable resolution of the infection.

Our patient had a medical history that might have predisposed him to such an infection. Years before the presentation, he had been referred for cardiac surgery due to severe ischemic heart disease. Aortic-coronary bypass surgery and replacement of the aortic valve were performed. Nevertheless, contrary to the literature cases, there was no postoperative complication after the cardiac surgery nor local deterioration during sternal wound healing. Furthermore, during a long time of follow up, no features of local infection were documented and thus no indications to the microbiological investigation were stated.

To the authors’ best knowledge, the presented case report is the second case of confirmed *Gordonia* infection in a patient with chronic obstructive pulmonary disease. Brust and co-workers [10] described a 78-year old female with COPD diagnosis receiving long term oxygen therapy, oral corticosteroids due to exacerbation and parenteral nutrition via a Hickman catheter. She experienced nausea, vomiting, diarrhoea and lethargy, with neutrophilic fever. The infection caused by *G. sputi* developed probably following the decreased immunocompetence status and catheter indwelling.

Although he suffered from chronic respiratory and cardiovascular diseases, our patient has not been an immunocompromised individual. He did not present general severe symptoms, but rather a prolonged lasting cough with expectoration of abundant purulent sputum, episodes of increased body temperature (up to 37.5 °C) and episodes of transient dyspnea, attributed to his coexisting diseases. Finally, besides brief oral trimethoprim-sulfamethoxazole therapy, he did not require further antimicrobial pharmacotherapy. The patient was referred for intensive physiotherapy and bronchial drainage. The remarkable improvement in the clinical status was evident, and a sputum expectoration almost wholly disappeared.

Researchers and the authors of the *Gordonia bronchialis* infection case reports highlighted the difficulties with the microbiological investigation. Although it is clear that identifying the pathogen is critical to ensuring that patients are correctly diagnosed and treated, the preliminary culture often revealed different bacteria before the final results: *Actinomycetes, Nocardia and Mycobacteria Other Than Tuberculosis.* Such findings are justified by the phylogenetic diversion of the order *Actinomycetales* that comprises various lung pathogens [23]. The authors indicated the methods that appeared to be essential in establishing the aetiology of infection. In most presented cases, the species identifications required the use of advanced techniques: PCR, 16s rRNA gene sequencing method and MALDI-TOF [3,21,24,25].

*Gordonia bronchialis* is Gram-positive, catalase-positive, weakly acid-fast, thinly beaded coccobacilli that does not produce aerial hyphae [26]. The isolation of *G. bronchialis* strains requires 3 to 4 days of incubation, although false-negative results and underestimations of infections caused by this species are not rare [27]. Therefore, *G. bronchialis* can be missed in clinical specimens if the incubation time is limited to less than 72 h [6]. Furthermore, this bacterium is difficult to identify at the genus and species level because *Gordonia* species require extensive biochemical and morphological testing. Moreover, phenotypic identification of *Gordonia* spp. may be inconclusive, and biochemical profiles can lead to incorrect identification of isolates as non-tuberculosis mycobacteria, *Nocardia* or other actinomycetes [24]. Therefore, the use of genotypic methods such as 16S rRNA gene sequencing [2] or advanced microbiology diagnosis techniques such as MALDI-TOF MS is needed to identify the bacterium to species level [24].

A diagnostic dilemma and lack of a precise diagnostic path were also our experience. The first preliminary diagnosis indicated NTM, but identification by genetic test did not confirm any species belonging to the Mycobacterium genus. Then, the new characterization, based on observed morphology, identified an aerobic actinomycete, possibly a *Nocardia* spp. Finally, the modified protocol for Mycobacterium and Nocardia identification was applied and this led to the identification of *Gordonia bronchialis*. The 16s rRNA gene sequencing method confirmed the result of the microbiological investigation.

## 5. Conclusions

Although *Gordonia* spp. are environmental bacteria, they are observed to be increasing implications in human infection aetiology. However, as the bacteria are ubiquitous in the environment, soil and water, the differentiation between environmental contamination and pathogenic meaning can be problematic and questionable. These difficulties may result in the underdiagnosing of *Gordonia* spp. infections [28].

What is worth underlining is that in almost all cited publications and reports, the authors highlighted the difficulties in the final identification of *Gordonia* species. Thus, this emerging pathogen requires extensive and contemporary microbiological diagnostics, including molecular identification on gene sequence. That takes us to a strong suggestion that the diagnostic path and microbiological investigation need to be performed very reliably and meticulously.

The assessment of the clinical significance of the *Gordonia* spp. is becoming increasingly relevant. The reports of *Gordonia* infections, especially in immunocompetent patients, are valuable as the source of knowledge on precise identification based on genomic sequencing. Consequently, a better understanding of these organisms could lead to their more frequent recognition as pathogens in the broader range of human diseases. The more reports about the difficult-to-diagnose *Gordonia* spp. the more knowledge and diagnostical experience that supports the diagnostic path recommendations. The presented case draws attention to mild *Gordonia* species infections, which may otherwise remain undiagnosed.

## Figures and Tables

**Table 1:** The identified species comparison to 16s ribosomal RNA database (Bacteria and Archaea) with Megablast (Optimize for highly similar sequences) in two of four samples of the analyzed PCR product.

**Sample 2**								
**L.P.**	**Description**	**Scientific Name**	**Max Score**	**Total Score**	**Query Cover**	**E Value**	**Per. Ident**	**Accession**
1	*Gordonia bronchialis* DSM 43247 16S ribosomal RNA, partial sequence	*Gordonia bronchialis* DSM 43247	586	586	100%	3.0 × 10^−167^	100.00%	NR_074529.1
2	*Gordonia bronchialis* DSM 43247 16S ribosomal RNA, partial sequence	*Gordonia bronchialis* DSM 43247	586	586	100%	3.0 × 10^−167^	100.00%	NR_027594.1
3	*Gordonia effusa* strain IFM 10200 16S ribosomal RNA, partial sequence	*Gordonia effusa*	580	580	100%	1.0 × 10^−165^	99.68%	NR_041008.1
4	*Gordonia bronchialis* DSM 43247 strain NCTC 10667 16S ribosomal RNA, partial sequence	*Gordonia bronchialis* DSM 43247	575	575	100%	6.0 × 10^−164^	99.37%	NR_119065.1
5	*Gordonia soli* strain CC-AB07 16S ribosomal RNA, partial sequence	*Gordonia soli*	575	575	100%	6.0 × 10^−164^	99.37%	NR_043331.1
6	*Gordonia rubripertincta* strain ATCC 14352 16S ribosomal RNA, partial sequence	*Gordonia rubripertincta*	569	569	100%	3.0 × 10^−162^	99.05%	NR_119117.1
7	*Gordonia westfalica* strain Kb2 16S ribosomal RNA, partial sequence	*Gordonia westfalica*	569	569	100%	3.0 × 10^−162^	99.05%	NR_025468.1
8	*Gordonia rubripertincta* strain N4 16S ribosomal RNA, partial sequence	*Gordonia rubripertincta*	569	569	100%	3.0 × 10^−162^	99.05%	NR_104572.1
9	*Gordonia namibiensis* strain NAM-BN063A 16S ribosomal RNA, partial sequence	*Gordonia namibiensis*	569	569	100%	3.0 × 10^−162^	99.05%	NR_025165.1
10	*Gordonia hankookensis* strain ON-33 16S ribosomal RNA, partial sequence	*Gordonia hankookensis*	569	569	100%	3.0 × 10^−162^	99.05%	NR_104507.1
**Sample 3**								
**L.P.**	**Description**	**Scientific Name**	**Max Score**	**Total Score**	**Query Cover**	**E Value**	**Per. Ident**	**Accession**
1	*Gordonia bronchialis* DSM 43247 16S ribosomal RNA, partial sequence	*Gordonia bronchialis* DSM 43247	575	575	100%	5.0 × 10^−164^	100.00%	NR_074529.1
2	*Gordonia bronchialis* DSM 43247 16S ribosomal RNA, partial sequence	*Gordonia bronchialis* DSM 43247	575	575	100%	5.0 × 10^−164^	100.00%	NR_027594.1
3	*Gordonia effusa* strain IFM 10200 16S ribosomal RNA, partial sequence	*Gordonia effusa*	569	569	100%	3.0 × 10^−162^	99.68%	NR_041008.1
4	*Gordonia bronchialis* DSM 43247 strain NCTC 10667 16S ribosomal RNA, partial sequence	*Gordonia bronchialis* DSM 43247	564	564	100%	1.0 × 10^−160^	99.36%	NR_119065.1
5	*Gordonia soli* strain CC-AB07 16S ribosomal RNA, partial sequence	*Gordonia soli*	564	564	100%	1.0 × 10^−160^	99.36%	NR_043331.1
6	*Gordonia rubripertincta* strain ATCC 14352 16S ribosomal RNA, partial sequence	*Gordonia rubripertincta*	558	558	100%	6.0 × 10^−159^	99.04%	NR_119117.1
7	*Gordonia westfalica* strain Kb2 16S ribosomal RNA, partial sequence	*Gordonia westfalica*	558	558	100%	6.0 × 10^−159^	99.04%	NR_025468.1
8	*Gordonia rubripertincta* strain N4 16S ribosomal RNA, partial sequence	*Gordonia rubripertincta*	558	558	100%	6.0 × 10^−159^	99.04%	NR_104572.1
9	*Gordonia namibiensis* strain NAM-BN063A 16S ribosomal RNA, partial sequence	*Gordonia namibiensis*	558	558	100%	6.0 × 10^−159^	99.04%	NR_025165.1
10	*Gordonia hankookensis* strain ON-33 16S ribosomal RNA, partial sequence	*Gordonia hankookensis*	558	558	100%	6.0 × 10^−159^	99.04%	NR_104507.1

## Data Availability

The clinical data of the patient are available in the hospital database.
